# ID 292 - Eficácia, Segurança e Impacto Orçamentário da Oxicodona Associada com Naloxona no Controle da Dor no Pós-Operatório de Cirurgias Ortopédicas

**DOI:** 10.5327/2237-9622.2025.v34s1.292

**Published:** 2025-11-25

**Authors:** Cristiane Rocha de Oliveira, Grasiela Martins da Silva, Quenia Cristina Dias Morais, Verônica Clemente

**Keywords:** oxicocona, naloxona, combinação oxicodona e naloxona, procedimento cirúrgico ortopédico

## Abstract

**Introdução:**

A analgesia nas quatro primeiras semanas do pós-operatório (PO) de cirurgias ortopédicas é um importante fator para recuperar a mobilidade do paciente. A oxicodona (OX) é um agonista opioide, com efeito analgésico, sedativo e ansiolítico. A naloxona (N) é um antagonista que neutraliza a constipação induzida pelos opioides por competir pelos receptores intestinais. A associação OXN (Targin®) é apresentada na forma de comprimidos de ação prolongada com liberação por 12 horas. É indicado no PO quando já iniciado no pré-operatório e mediante a previsão médica de que a dor será moderada a intensa por tempo prolongado. A incorporação da OXN no Instituto Nacional de Traumatologia e Ortopedia Jamil Haddad (Into) foi demandada como alternativa para o tratamento da dor moderada a intensa no PO de cirurgias ortopédicas de grande porte, com possíveis vantagens quanto à via de administração oral, constância nas concentrações plasmáticas, liberação para uso domiciliar, redução do tempo de internação e re-hospitalização pela constipação. Objetivos: avaliar a eficácia e a segurança da OXN de liberação prolongada no controle da dor dos pacientes adultos submetidos à cirurgias ortopédicas de grande porte; estimar o impacto orçamentário da incorporação para o Into.

**Método:**

Foram realizadas todas as etapas de uma revisão sistemática com busca nas bases MEDLINE (PubMed), Embase e Cochrane Library com vocabulários controlados. As 605 referências identificadas foram selecionadas e avaliadas por pares de revisores independentes, com divergências resolvidas por terceiro revisor. Critérios de inclusão:estudos primários ou de síntese avaliando a eficácia e a segurança da intervenção no cenário proposto. Critério de exclusão: OXN de liberação imediata. A avaliação da qualidade da evidência foi realizada com a ferramenta RoB2 e a certeza nos resultados com o sistema GRADE (do inglês, ). Para o impacto orçamentário (IO) sob a perspectiva Into em horizonte de cinco anos, foi utilizado custo direto de aquisição baseado no menor custo na Câmara de Regulação do Mercado de Medicamentos (Cmed). A estimativa da população se deu por demanda aferida. Foi considerada posologia e tempo de tratamento descrito em bula com difusão de 10%, 50% e de 100% nos três últimos anos.

**Resultados:**

Incluído um ensaio clínico randomizado (ECR) não cego que avaliou o controle da dor no PO de artroplastia total do joelho (ATJ) em 112 pacientes, 57 OXN e 55 morfina intravenosa em bomba infusora (MBI). Mensuração nas 48 horas de PO por escore médio de dor em repouso e flexão do joelho (0 = sem dor e 10 =p ior dor). Foram desfechos secundários: consumo equivalente de morfina e ocorrência de náuseas/vômitos (0 = sem náuseas, 1 = náusea leve e antiemético, 2 = vômitos). No primeiro e no segundo dia de PO, a OXN apresentou melhor controle da dor em repouso (0,89 ±1,54 versus 1,27 ±1,85, P=0,0019) e (1,03 ±1,69 versus 1,65 ±2,05, P=0,0006). Não houve diferenças estatisticamente significativas entre os grupos para dor em flexão e desfechos secundários. A evidência apresentou alto risco de viés e baixa certeza nos resultados. Esquemas posológicos de 7 e 14 dias para população de 100 pacientes/ano geram IO, em 5 anos, de R$ 28.828,80 e R$ 57.657,60. Pressuposto de 50% da população para cada esquema gera IO de R$ 43.243,20.

**Conclusão:**

A OXN mostrou eficácia no controle da dor no PO de cirurgia de ATJ quando comparado com MBI, não sendo superior quanto aos demais desfechos. A evidência disponível não avaliou constipação, tempo de internação ou re-hospitalização. As limitações metodológicas do estudo devem ser consideradas para tomada de decisão. Se incorporada, monitoramento e estudos comparativos de eficácia e segurança devem ser planejados.

